# The soluble guanylyl cyclase stimulator BAY 41-2272 increases differentiation and function of brown adipocytes

**DOI:** 10.1186/2050-6511-14-S1-P20

**Published:** 2013-08-29

**Authors:** Jennifer Etzrodt, Linda S Hoffmann, Andreas Friebe, Alexander Pfeifer

**Affiliations:** 1 Institute for Pharmacology and Toxicology, University of Bonn, 53105 Bonn, Germany; 2 Physiologisches Institut, University of Würzburg, 97070 Würzburg, Germany

## Background

There are two types of adipose tissue. White adipose tissue (WAT) stores energy in the form of lipids. Brown adipose tissue (BAT) consumes energy to produce heat by thermogenesis, which depends on the brown adipocytes specific marker uncoupling protein-1 (UCP—1). Metabolically active BAT is present in adult humans and its energy consuming properties could be exploited to increase energy expenditure. We and others showed that the nitric oxide (NO)/ cyclic guanosine monophosphate (cGMP) pathway is crucial for brown adipocyte differentiation [1,2]. Here we studied the role of sGC in brown adipocyte differentiation employing the sGC stimulator BAY 41—2272.

## Material and methods

Mesenchymal stem cells isolated from BAT of newborn mice were differentiated into brown adipocytes *in vitro* using established protocols [1]. During differentiation, cells were incubated with 3 µM BAY 41—2272, 200 µM 8-pCPT-cGMP or 30 µM ODQ. Accumulated intracellular lipids were assessed by RedO staining and measurement of triglyceride (TG) content. To investigate the adipogenic program, Western Blot analysis of PPARγ, C/EBPα and AP2 was performed. Thermogenic differentiation of the cells was determined by protein expression analysis of Cytc and gene expression analysis of thermogenic markers *UCP—1*, *Cidea* and *PGC-1α.*

## Results

BAY 41-2272 increased lipid accumulation as determined by RedO staining and TG content (1,44-fold) compared to control. Protein expression of the adipogenic marker proteins PPARγ (1,4-fold), C/EBPα (1,5-fold) and AP2 (1,6—fold) were increased after BAY 41-2272 incubation compared to control. Adipogenic markers PPARγ (2,3-fold), C/EBPα (1,8-fold) and AP2 (2,1-fold) were increased after cGMP incubation compared to control. In contrast, inhibition of sGC by ODQ decreased protein expression of PPARγ (50%), C/EBPα (29%) and AP2 (51%). The thermogenic marker genes *UCP-1* (5,38-fold), *Cidea* (2,44-fold) and *PGC-1α* (2,26-fold) increased upon incubation with BAY 41-2272 compared to control. cGMP increased RNA levels of thermogenic markers *UCP—1* (3,36-fold), *Cidea* (2,5-fold) and *PGC-1α* (2,50 fold). ODQ decreased the expression of *UCP-1* (64%), *Cidea* (74%) and *PGC—1α* (44%). Protein expression of the mitochondrial marker Cytc was increased upon incubation with BAY 41—2272 (1,6-fold) and cGMP (1,77-fold). Protein expression of Cytc was lower in brown adipocytes incubated with ODQ (49%).

## Conclusion

Taken together, the results show that the sGC stimulator BAY 41—2272 increases the adipogenic and thermogenic program of brown adipocytes to a similar extent as the cGMP analogue. This hints to a therapeutic potential of BAY 41—2272. Its beneficial effect on adipogenesis and thermogenesis could lead to increased energy expenditure and could combat overweight and obesity.
